# Gene Expression-Related Changes in Morphologies of Organelles and Cellular Component Organization in Mucopolysaccharidoses

**DOI:** 10.3390/ijms22052766

**Published:** 2021-03-09

**Authors:** Lidia Gaffke, Karolina Pierzynowska, Estera Rintz, Zuzanna Cyske, Izabela Giecewicz, Grzegorz Węgrzyn

**Affiliations:** Department of Molecular Biology, University of Gdansk, Wita Stwosza 59, 80-308 Gdansk, Poland; lidia.gaffke@ug.edu.pl (L.G.); karolina.pierzynowska@ug.edu.pl (K.P.); estera.rintz@ug.edu.pl (E.R.); zuzanna.cyske@phdstud.ug.edu.pl (Z.C.); izabelagiecewicz@gmail.com (I.G.)

**Keywords:** mucopolysaccharidoses, transcriptomic analyses, organelles, electron microscopy

## Abstract

Mucopolysaccharidoses (MPS) are inherited metabolic diseases characterized by accumulation of incompletely degraded glycosaminoglycans (GAGs) in lysosomes. Although primary causes of these diseases are mutations in genes coding for enzymes involved in lysosomal GAG degradation, it was demonstrated that storage of these complex carbohydrates provokes a cascade of secondary and tertiary changes affecting cellular functions. Potentially, this might lead to appearance of cellular disorders which could not be corrected even if the primary cause of the disease is removed. In this work, we studied changes in cellular organelles in MPS fibroblasts relative to control cells. All 11 types and subtypes of MPS were included into this study to obtain a complex picture of changes in organelles in this group of diseases. Two experimental approaches were employed, transcriptomic analyses and electron microscopic assessment of morphology of organelles. We analyzed levels of transcripts of genes grouped into two terms included into the QuickGO database, ‘Cellular component organization’ (GO:0016043) and ‘Cellular anatomical entity’ (GO:0110165), to find that number of transcripts with significantly changed levels in MPS fibroblasts vs. controls ranged from 109 to 322 (depending on MPS type) in GO:0016043, and from 70 to 208 in GO:0110165. This dysregulation of expression of genes crucial for proper structures and functions of various organelles was accompanied by severe changes in morphologies of lysosomes, nuclei, mitochondria, Golgi apparatus, and endoplasmic reticulum. Interestingly, some observed changes occurred in all/most MPS types while others were specific to particular disease types/subtypes. We suggest that severe changes in organelles in MPS cells might arise from dysregulation of expression of a battery of genes involved in organelles’ structures and functions. Intriguingly, normalization of GAG levels by using recombinant human enzymes specific to different MPS types corrected morphologies of some, but not all, organelles, while it failed to improve regulation of expression of selected genes. These results might suggest reasons for inability of enzyme replacement therapy to correct all MPS symptoms, particularly if initiated at advanced stages of the disease.

## 1. Introduction

Lysosomal storage diseases (LSD) group over 50, out of several thousand known [1], monogenic disorders. Although single gene defects are defined in each such disease, it appears that we are still far from understanding molecular mechanisms leading to expression of complicated symptoms due to dysfunction of just one *locus*, which can be exemplified by unexpected phenotypes of persons bearing known pathogenic mutations [2]. On the other hand, LSD are often considered model genetic disorders as they are undoubtedly in the forefront of inherited disorders for which principles of molecular mechanisms were described, and for which molecular therapies became available [3]. 

Among all LSD, 11 diseases belong to mucopolysaccharidoses (MPS) which are caused by mutations in genes coding for lysosomal enzymes required for degradation of glycosaminoglycans (GAG) [4]. Accumulation of GAGs in lysosomes is the primary cause of MPS, and due to continuous production of these compounds and their impaired degradation, these diseases are progressive and severe, with expected average life span between one and two decades [4]. Unfortunately, despite incredible progress of molecular genetic studies and extensive medical investigations, only a few therapeutic options are available for MPS patients while none of them being able solve all, or even most, clinical problems [5,6]. 

Classification of MPS is based on the kind of stored GAG (dermatan sulfate (DS), heparan sulfate (HS), keratan sulfate (KS), chondroitin sulfate (CS), and hyaluronic acid) and the nature of deficient lysosomal enzyme [4]. Basic characterization of MPS types and subtypes is shown in Supplementary Table S1. 

Molecular and cellular biology studies indicated that although GAG storage is the primary cause of MPS, there are many secondary and tertiary cellular processes that are significantly changed in cells of the patients relative to healthy persons (summarized and discussed in [7]). Such changes may arise from accumulation of dysfunctions of proteins and/or genes appearing in the course of the disease and can cause further dysregulations. Potentially, this might lead to appearance of cellular disorders which could not be corrected even if the primary cause of the disease is removed. Such a hypothesis might explain a failure in attempts to correct all or most MPS symptoms by any treatment employed so far, as previously established cellular dysfunctions could not be reversed by simple normalization of GAG levels [7]. Recent investigations indicated that secondary and tertiary changes in MPS could be caused by dysregulations of many genes. The changes reported to date include either significantly elevated or decreased levels of transcripts of genes coding for proteins involved in behavioral regulation [8] and cellular processes [9], including apoptosis [10] and cell activation [11]. Those transcriptomic studies revealed that expression of hundreds of genes is changed in various MPS types relative to normal (control) cell lines. Interestingly, in vast majority of cases, particular genes were either down- or upregulated in majority, if not all, MPS types and subtypes, indicating that the processes controlling transcription might be common in different MPS diseases, though significant differences between MPS types were also noted [8,9,10,11].

Knowing that structures of cellular organelles are changed in MPS [4,12], in this work we asked if these changes can be caused by dysregulation of genes related to their formation and functions. Transcriptomic and microscopic studies, accompanied with biochemical determination of GAG levels and Western-blot analyses of abundance of selected proteins, were conducted to address this research hypothesis. In addition, to assess principles of molecular mechanisms of changes in organelles in MPS, effects of decreasing GAG levels by using recombinant human enzymes, a-L-iduronidase and iduronate sulfatase, in MPS I and MPS II, respectively (these enzymes are deficient in corresponding MPS types and their recombinant forms are used in enzyme replacement therapy for patients), on morphologies of organelles and levels of selected proteins were tested. 

## 2. Results

**Figure 1:**
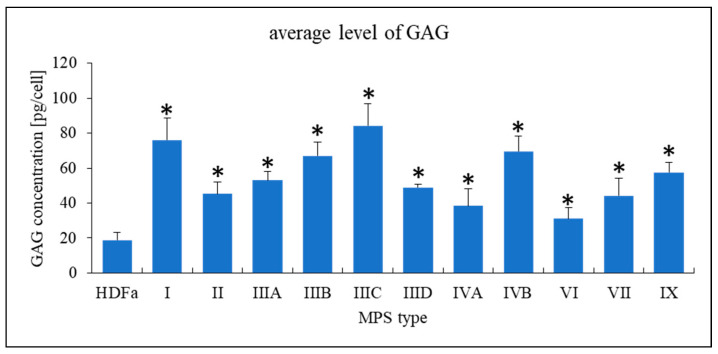
Levels of GAGs in tested lines of MPS fibroblasts relative to the control cell line (HDFa). The presented values are mean values from three independent experiments with error bars representing SD. Statistically significant differences (*; *p* < 0.05) were found for each MPS type relative to HDFa.

In our studies, we used fibroblasts derived from patients suffering from all types and subtypes of MPS. These patients were characterized by severe phenotypes which corresponded to mutations in their cells (where relevant data were available) and/or a lack of detectable deficient enzyme activity (Supplementary Table S2). We confirmed that, as expected, levels of GAGs accumulated in tested MPS fibroblasts were significantly higher than that determined for the HDFa control cell line (Figure 1). These results are compatible with the previously performed genetic and biochemical characterization of these cell lines, as reported in Supplementary Table S2. 

Using transcriptomic data, we analyzed levels of transcripts of genes grouped into two terms defined by the Gene Ontology Consortium and included into the QuickGO database, ‘Cellular component organization’ (GO:0016043) and ‘Cellular anatomical entity’ (GO:0110165). When assessing following parameters: false discovery rate (FDR) <0.1 and *p* < 0.1 (these parameters were chosen according to previously published reports [8,9,10,11]), we found that number of transcripts with significantly changed levels in MPS fibroblasts vs. HDFa controls was relatively high, ranging from 109 to 322 (depending on MPS type) in GO:0016043, and from 70 to 208 in GO:0110165 (Table 1). 

High numbers of transcripts related to cellular component organization (GO:0016043) and cellular anatomical entity (GO:0110165) that were up- or downregulated in MPS cells (Table 1) encouraged us to analyze expression of corresponding genes in more detail. Therefore, we divided the genes according to the QuickGO database, employing child terms as defined in this database and presented in Supplementary Figure S1. 

**Figure 2:**
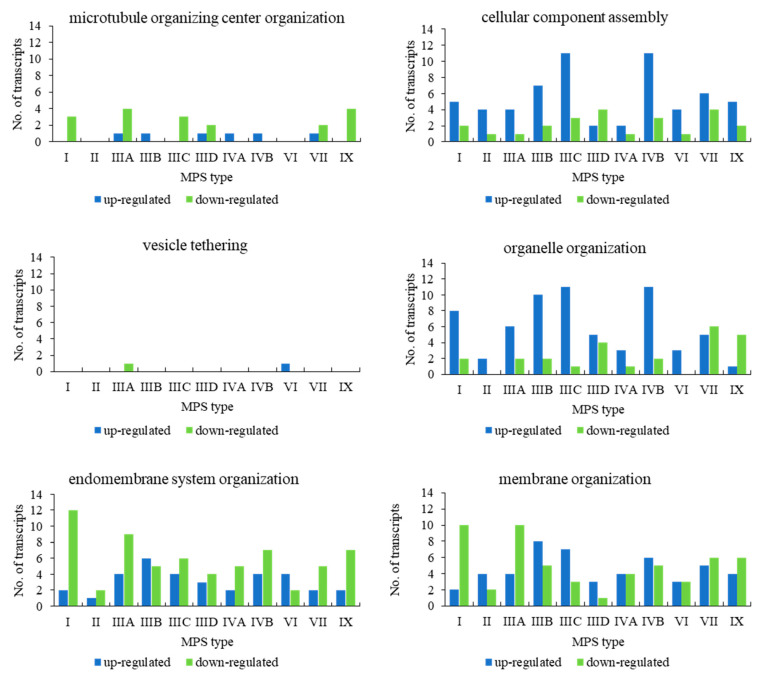
Number of up- and downregulated transcripts (at FDR < 0.1; *p* < 0.1) with division into selected processes (child terms of ‘cellular component organization’) in different MPS types relative to control cells (HDFa).

When assessing genes grouped in the child terms of cellular component organization (GO:0016043), i.e., microtubule organizing center organization (GO:0031023), cellular component assembly (GO:0022607), vesicle tethering (GO:0099022), organelle organization (GO:0006996), endomembrane system organization (GO:0010256), and membrane organization (GO:0061024), we found a significant number of transcripts whose levels were either elevated or decreased in MPS cells in all types of the disease in almost all child terms (Figure 2). The exception was vesicle tethering (GO:0099022), in which only one transcript was significantly changed (relative to the control) in MPS IIIA and one in VI (with no changes in other types). Moreover, in microtubule organizing center organization (GO:0031023), only a few genes were up- and/or downregulated, with MPS II and VI types in which no changes could be detected (Figure 2). 

**Figure 3:**
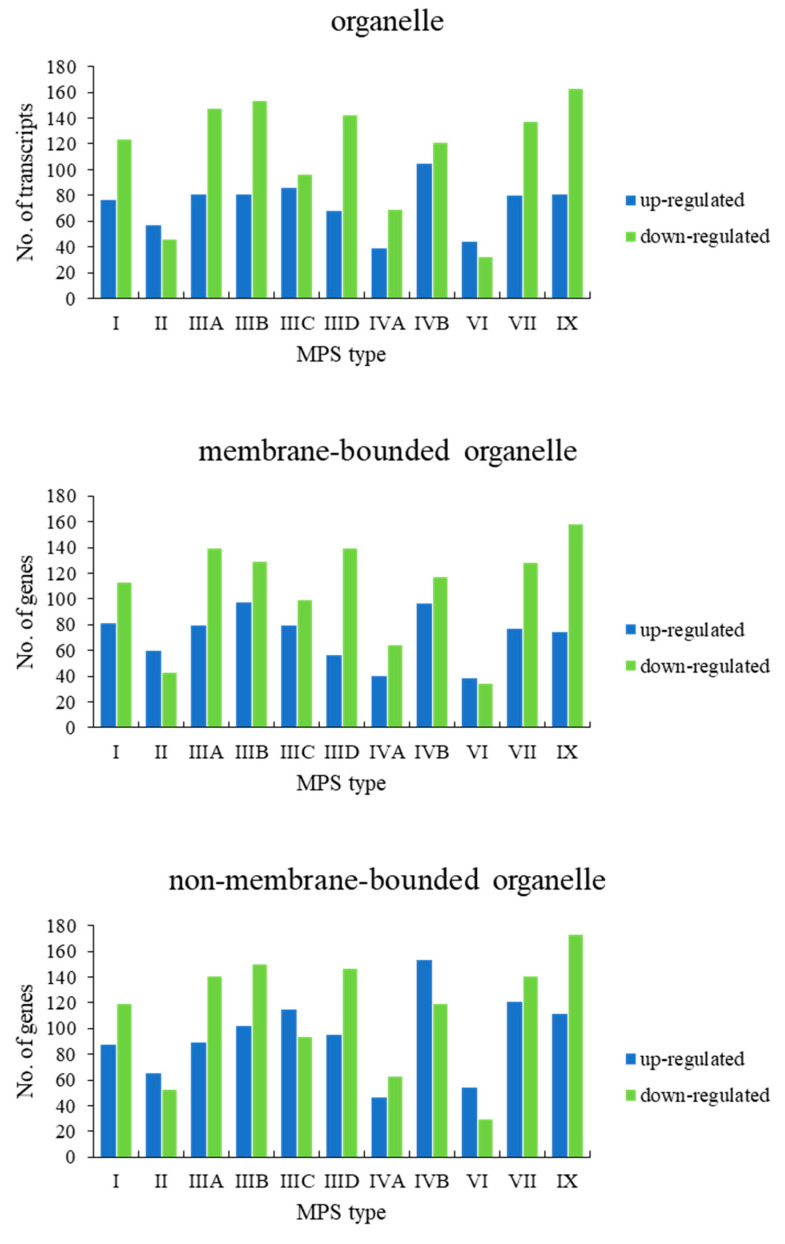
Number of up- and downregulated transcripts (at FDR < 0.1; *p* < 0.1) with division into selected organelles (child terms of ‘cellular anatomical entity) in different MPS types relative to control cells (HDFa).

**Figure 4:**
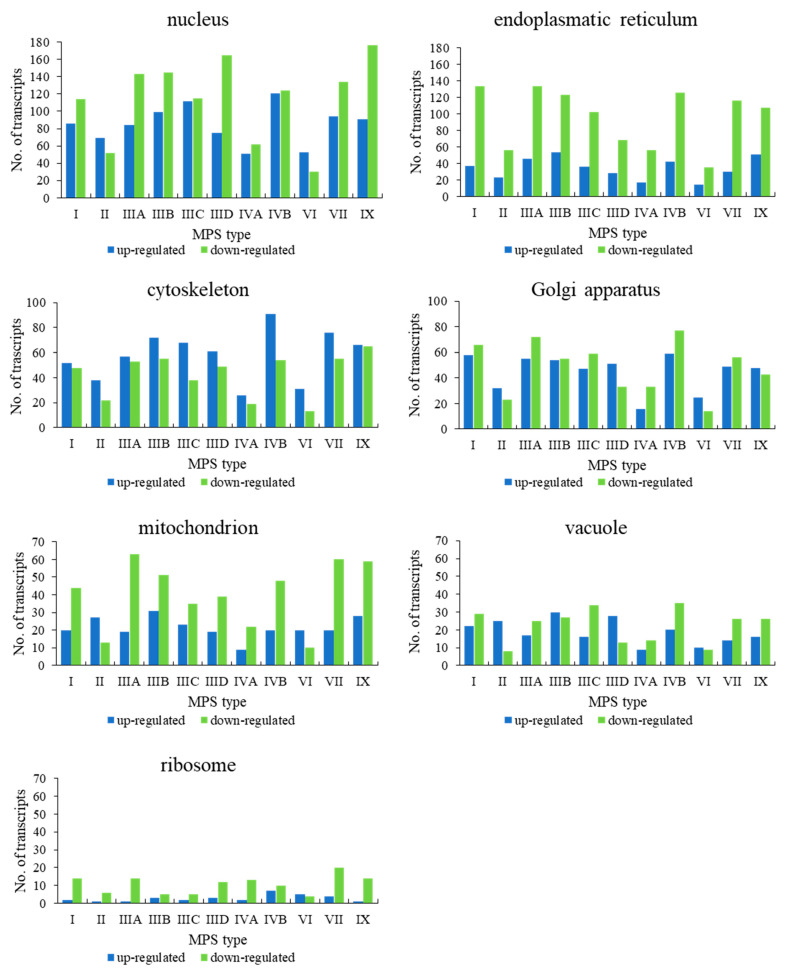
Number of up- and down-regulated transcripts (at FDR < 0.1; *p* < 0.1) with division into selected organelles (child terms of ‘intracellular membrane-bounded organelle’ (GO:0043231) and ‘intracellular non-membrane-bounded organelle’ (GO:0043232)) in different MPS types relative to control cells (HDFa).

When assessing genes grouped in the child terms of cellular anatomical entity (GO:0110165), i.e., membrane-bounded organelle (GO:0043227) and non-membrane-bounded organelle (GO:0043228), a significant number of genes revealed changed expression in all MPS types (Figure 3). Therefore, we divided these processes to lower levels child terms, representing different organelles. Again, in all cases, we observed a significant number of changed genes in all types of MPS (Figure 4). These results suggested that dysmorphology of organelles in MPS cells may be related to gene expression regulation.

We asked what transcripts of genes related to organelles were changed in at least nine (out of 11) types/subtypes of MPS. Considering two terms, cellular component organization (GO:0016043) and cellular anatomical entity (GO:0110165), we identified several genes fulfilling this criterium (Table 2). Interestingly, all these genes were either up- or downregulated in all MPS types/subtypes. For the term ‘cellular component organization’, we identified *MFAP5*, *PFN1*, *CAPG*, and *POSTN* genes that were upregulated in all MPS cell lines, and *SIN3B* and *SAR1A* genes that were downregulated. For the term ‘cellular anatomical entity’, we identified *SH3BP5*, *POSTN*, and *ARSA* genes that were upregulated in all MPS cell lines, and *TMEM38B*, *EMP1*, and *ABHD5* genes that were downregulated. Such a common pattern of dysregulation of expression of different genes in all tested MPS cell lines (Table 2) suggests that similar, if not the same, control processes related to structures and functions of cellular organelles are impaired in all types and subtypes of MPS. 

To assess what genes are particularly strongly dysregulated in MPS cells, we assessed transcripts of levels changed over 16 times (log_2_FC > 4) in such cells relative to controls. Considering both analyzed GO terms related to organelles, cellular component organization (GO:0016043) and cellular anatomical entity (GO:0110165), we could identify 20 and 18 transcripts, respectively, whose levels were extremely (>16 times) changed in at least one MPS cell line relative the control HDFa line (Table 3). We observed that if any of these transcripts was changed in more than one MPS type/subtype, the direction of the change (up- or downregulation) was always the same in all MPS lines. Again, this strongly suggests the existence of common mechanisms of dysregulation of expression of various genes in all types/subtypes of MPS.

Knowing that expression of a relatively large number of genes related to organelles is changed in MPS fibroblasts, we investigated morphology of these cellular components in fibroblasts derived from patients suffering from all know MPS types and subtypes. Samples of cell cultures were prepared for electron microscopic analyses, and electron micrographs were investigated to assess morphology of lysosomes, nuclei, mitochondria, Golgi apparatus, and endoplasmic reticulum. At least 100 cells were investigated for each kind of organelle, and following measurement or assessment of specific features, statistical analyses were performed after comparison to results obtained for control fibroblasts.

**Figure 5:**
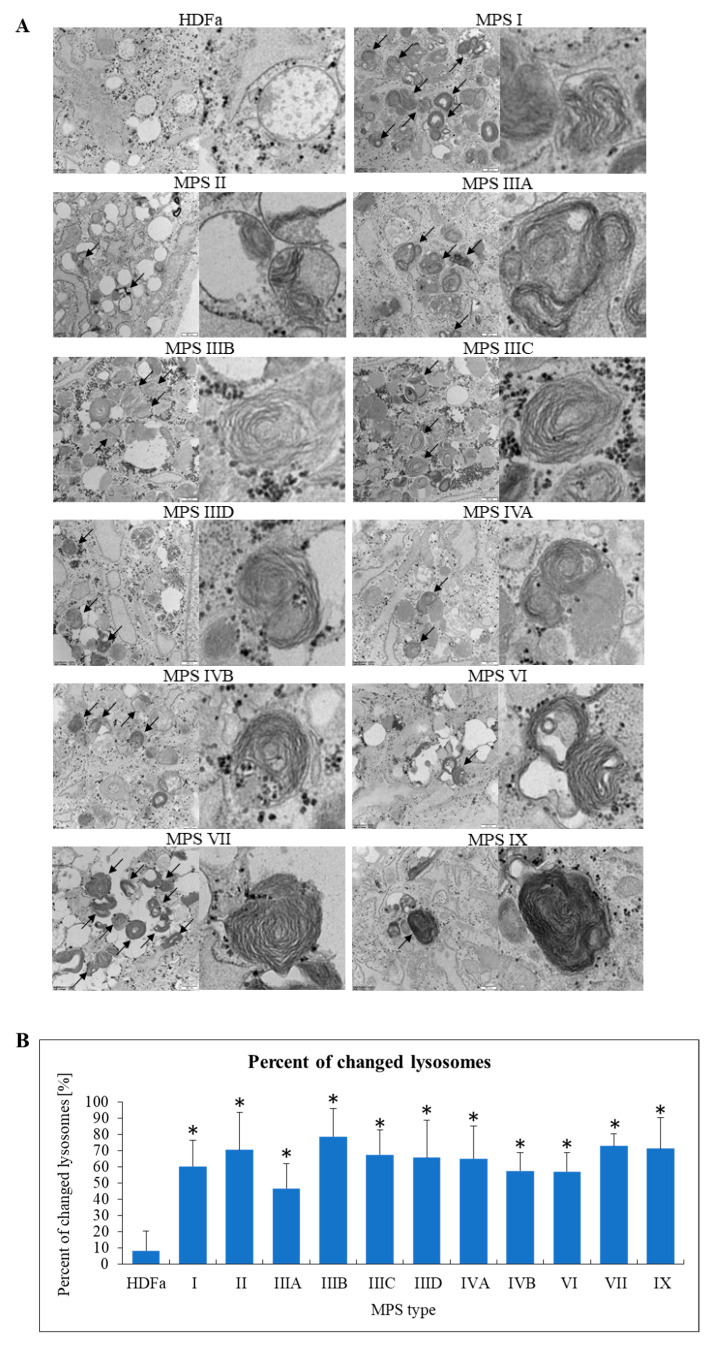
Morphology of lysosomes in MPS fibroblasts in comparison to control cells (HDFa cell line). Representative electron micrographs are presented in (**A**) (size bars represent 500 nm; arrows indicate changes in these structures). Quantification of changes in lysosomes is presented in (**B**). Mean values ± SD are presented, with asterisks representing statistically significant (*p* < 0.05) differences relative to the HDFa control.

Since MPS belong to lysosomal storage diseases (LDS), we started our analyses from assessment of changes in these organelles. In fact, lysosomal changes in various MPS types have been widely described previously [4]. Therefore, assessment of lysosomes was, on one hand, a test for reliability of our experimental system, but on the other hand, our analyses allowed to perform simultaneous analyses of lysosomal changes in all MPS types/subtypes. Characteristic inclusions in lysosomes were observed, along with specific structures described previously (see [4] for details) as ‘onion skin’ and ‘zebra bodies’ (Figure 5A). These results confirmed that under experimental conditions used in our study, we were able to observe lysosomal changes described previously as characteristic for MPS. Moreover, we quantified our result by determining fractions (percentages) of changed lysosomes in all tested cell lines. In control cells, only a small percent of lysosomes revealed abnormalities in morphology, while in each MPS type/subtype the fraction of changes lysosomes was 45% (in MSP IIIA) or higher (Figure 5B).

**Figure 6:**
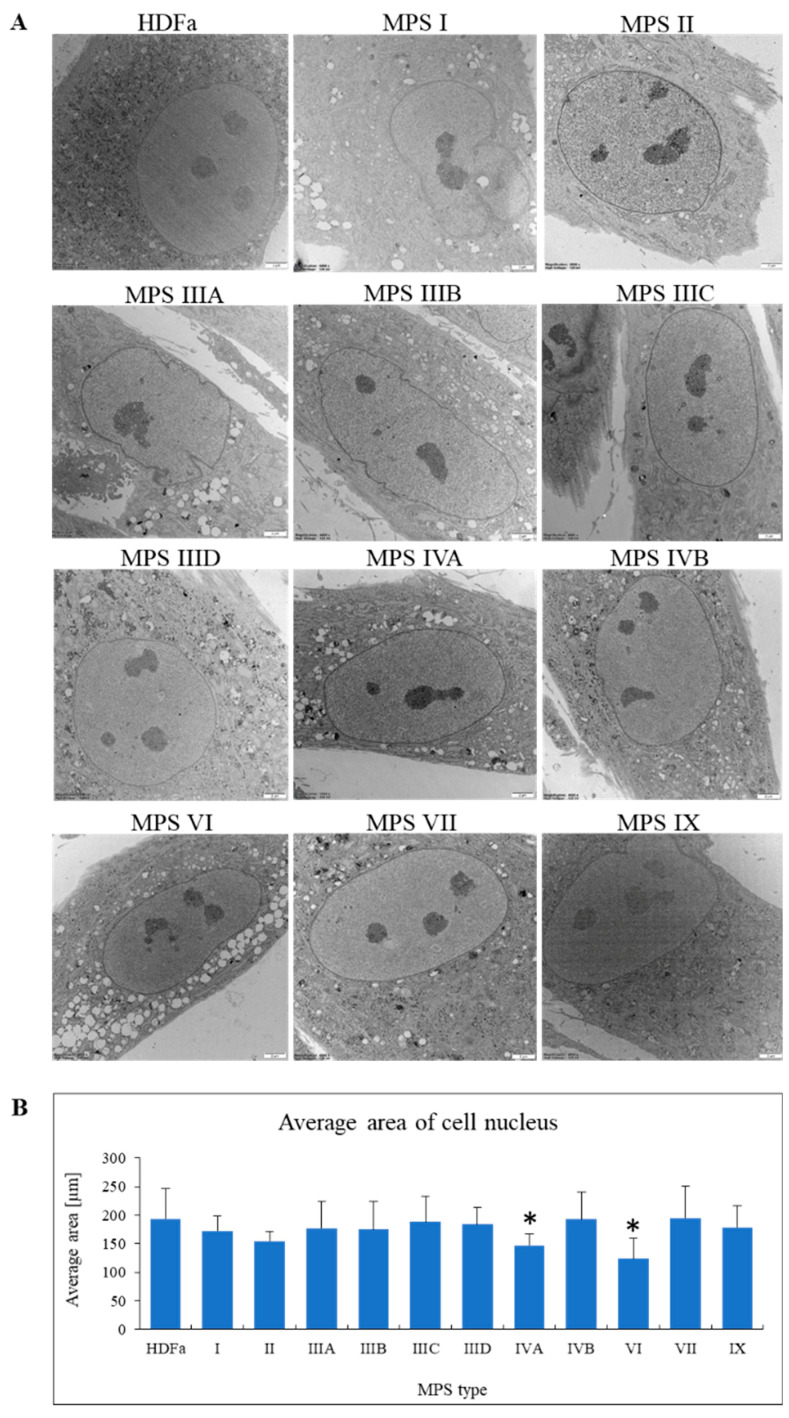
Morphology of nuclei in MPS fibroblasts in comparison to control cells (HDFa cell line). Representative electron micrographs are presented in (**A**) (size bars represent 2 mm). Quantification of changes in area of nucleus is presented in (**B**). Mean values ± SD are presented, with asterisks representing statistically significant (*p* < 0.05) differences relative to the HDFa control.

After confirming that morphology of lysosomes is severely changed in all MPS cell lines, we investigated other organelles. When assessing cellular nuclei, no specific abnormalities or characteristic structures were found in MPS fibroblasts relative to control cells (Figure 6A). However, when measuring size of the nucleus, we detected some differences. Since the majority of nuclei of fibroblasts in our experiments appeared under electron microscope as elliptical structures when observing cross-sections of cells, we measured the area of each nucleus. Under these conditions, nuclei of MPS type IVA and VI appeared smaller than those in control cells, while no such differences could be detected in other MPS types (Figure 6B). 

**Figure 7:**
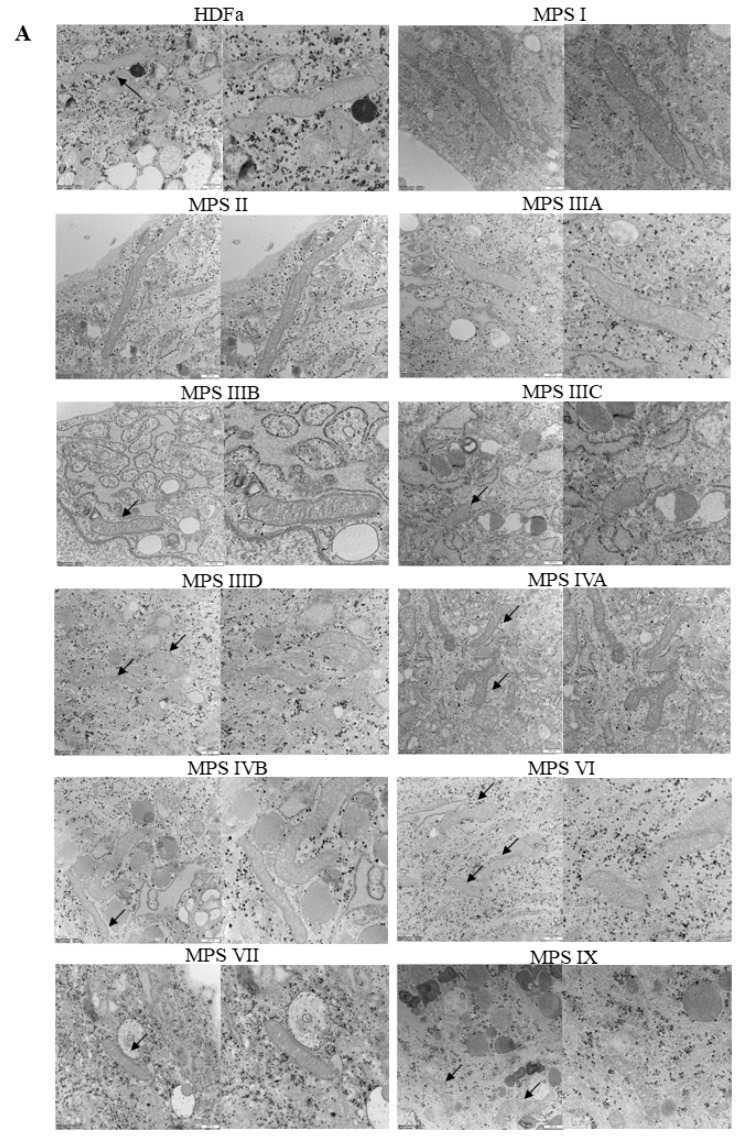

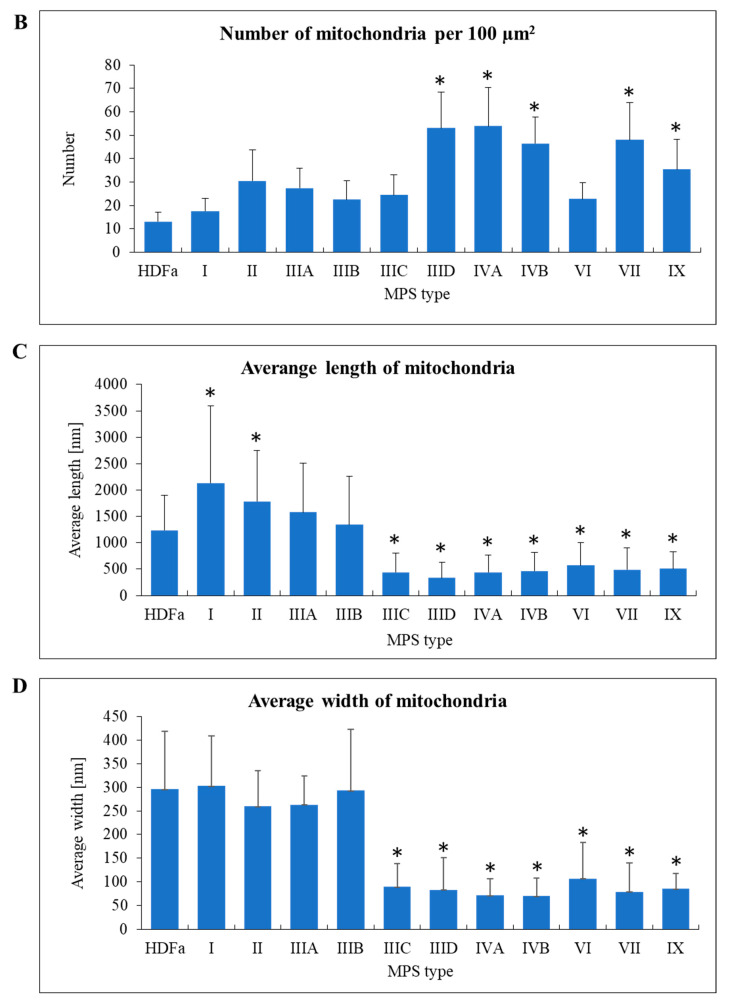
Morphology of mitochondria in MPS fibroblasts in comparison to control cells (HDFa cell line). Representative electron micrographs are presented in (**A**) (size bars represent 500 nm; arrows indicate mitochondria). Quantification of changes in mitochondria is presented in (**B**–**D**), indicating their number (**B**), length (**C**) and width (**D**). Mean values ± SD are presented, with asterisks representing statistically significant (*p* < 0.05) differences relative to the HDFa control.

When assessing mitochondria, we calculated their number per area of cell on electron micrograph, their length and width (Figure 7A). Interestingly, mitochondria were significantly more abundant in MPS IIID, IVA, IVB, VII, and IX fibroblasts than in control cells and in other MPS types (Figure 7B). On the other hand, mitochondria in MPS I and II were longer than in control cells, while those in MPS IIIC, IIID, IVA, IVB, VI, VII, and IX were shorter than in fibroblasts derived from a healthy person (Figure 7C). Moreover, width of these organelles was smaller in MPS IIIC, IIID, IVA, IVB, VI, VII, and IX, relative to the HDFa control and other MPS types (Figure 7D). These results indicate an interesting correlation that in some MPS types the abundance of mitochondria in cells is higher than in control cells, while these mitochondria appear shorter and thinner.

**Figure 8:**
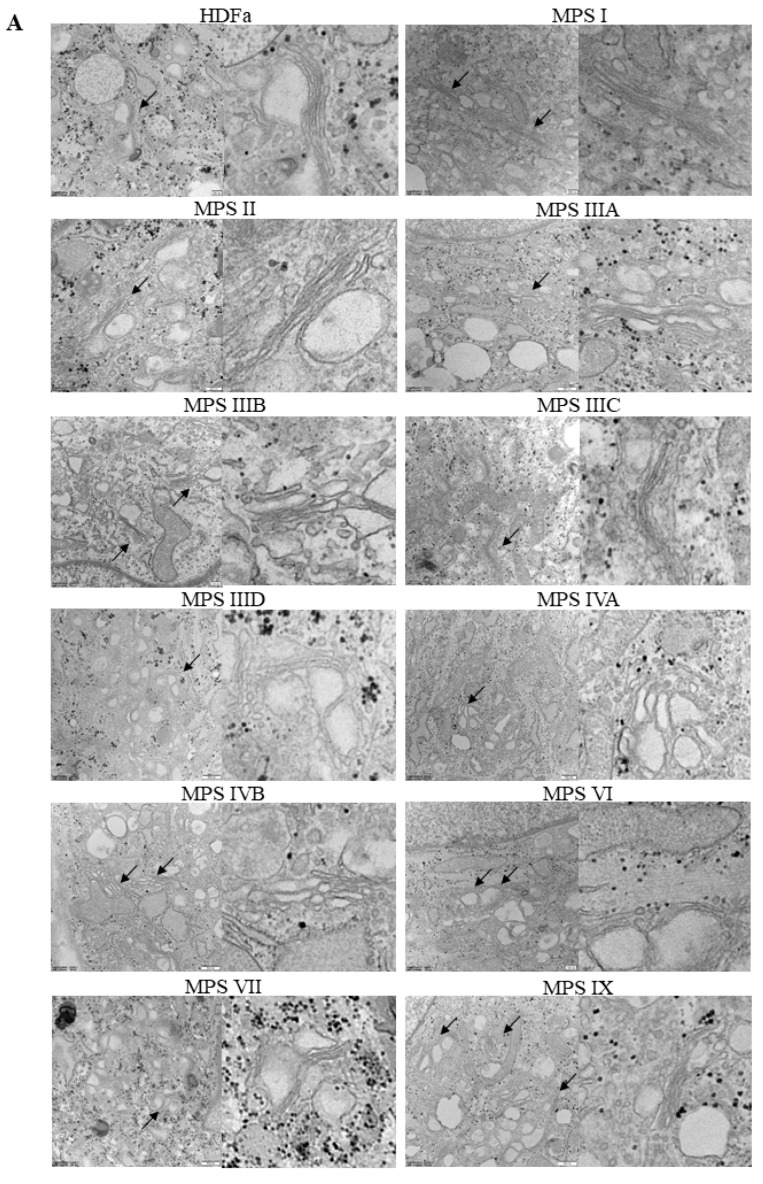

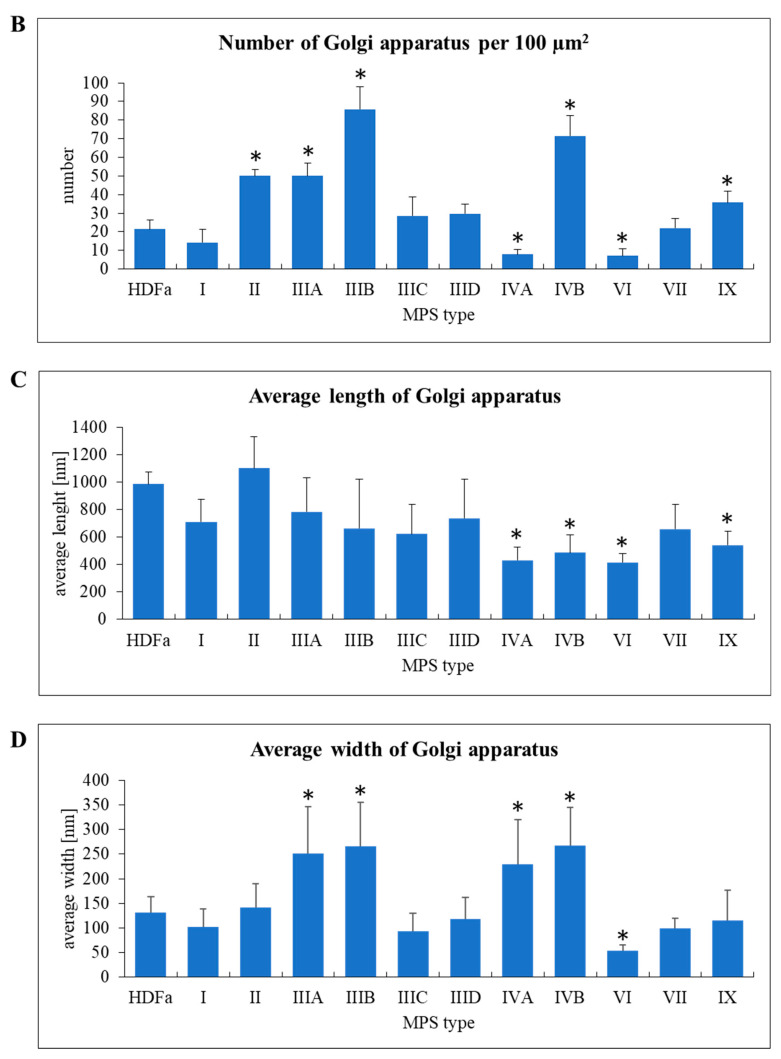
Morphology of Golgi apparatus in MPS fibroblasts in comparison to control cells (HDFa cell line). Representative electron micrographs are presented in (**A**) (size bars represent 200 nm; arrows indicate Golgi structures). Quantification of changes in Golgi structures is presented in (**B**–**D**), indicating their number (**B**), length (**C**) and width (**D**). Mean values ± SD are presented, with asterisks representing statistically significant (*p* < 0.05) differences relative to the HDFa control.

Similar parameters were measured in Golgi apparatus, despite a significantly different morphology of these organelles in comparison to mitochondria (Figure 8A). Huge variability in abundance of mitochondria was observed between different MPS types. In MPS II, IIIA, IIIB, IVB, and IX the number of these organelles was significantly higher than in control cells when calculated per area of cross-sectioned cells, while in MPS IVA and VI Golgi apparatus was less abundant (Figure 8B). In MPS IVA, IVB, VI, and IX we observed “fragmentation” of Golgi ribbons which appeared significantly shorter than in HDFa (Figure 8C). On the other hand, in MPS IIIA, IIIB, IVA, and IVB the ribbons were significantly wider (Figure 8D). 

**Figure 9:**
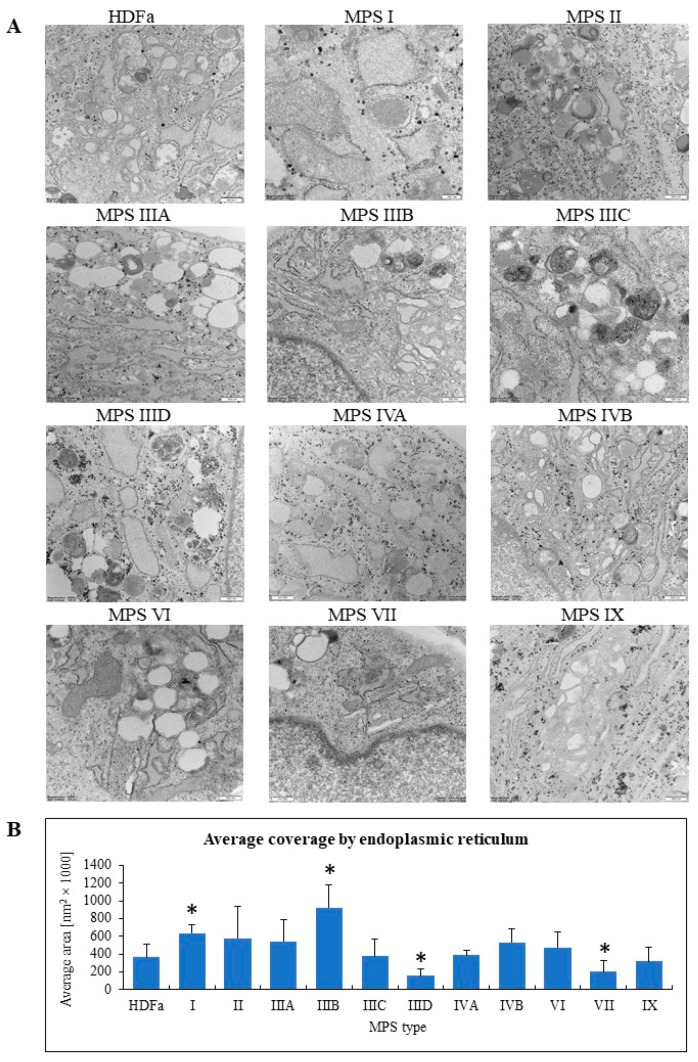
Morphology of endoplasmic reticulum in MPS fibroblasts in comparison to control cells (HDFa cell line). Representative electron micrographs are presented in (**A**) (size bars represent 500 nm). Quantification of changes in endoplasmic reticulum is presented in (**B**). Mean values ± SD are presented, with asterisks representing statistically significant (*p* < 0.05) differences relative to the HDFa control.

When considering endoplasmic reticulum, we assessed area covered by this cellular structure to find that it was bigger in MPS I and IIIB, and smaller in MPS IIID and VII (Figure 9).

**Figure 10:**
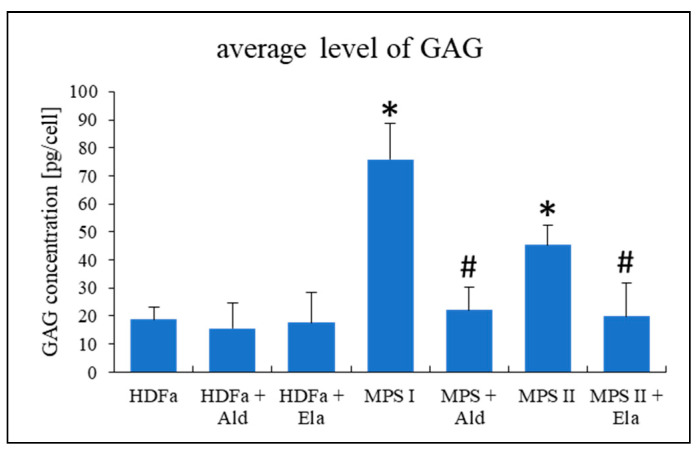
Levels of GAGs in HDFa (control) cells and MPS I and MPS II fibroblasts with and without treatment with 0.58 mg/L α-L-iduronidase (Aldurazyme, Ald) or 0.5 mg/L iduronate sulfatase (Elaprase, Ela) for 24 h. Mean values ± SD are presented, with asterisks and hashtags representing statistically significant (*p* < 0.05) differences relative to the HDFa control and untreated MPS, respectively.

In the next step, we asked whether supplementation of cultures of MPS I and MPS II fibroblasts with enzymes that are otherwise deficient in these cells (such enzymes are commercially available due to established enzyme replacement therapies) can restore morphologies of different cellular organelles. First, we tested if 24-h incubation of cells with the recombinant enzymes (0.58 mg/L of α-L-iduronidase, Alurazyme; 0.5 mg/L of iduronate sulfatase, Elaprase; the doses were identical to those used in enzyme replacement therapies) can correct levels of GAGs in tested MPS cells. We found that such a treatment was sufficient to correct GAG amounts in cells (Figure 10).

**Figure 11:**
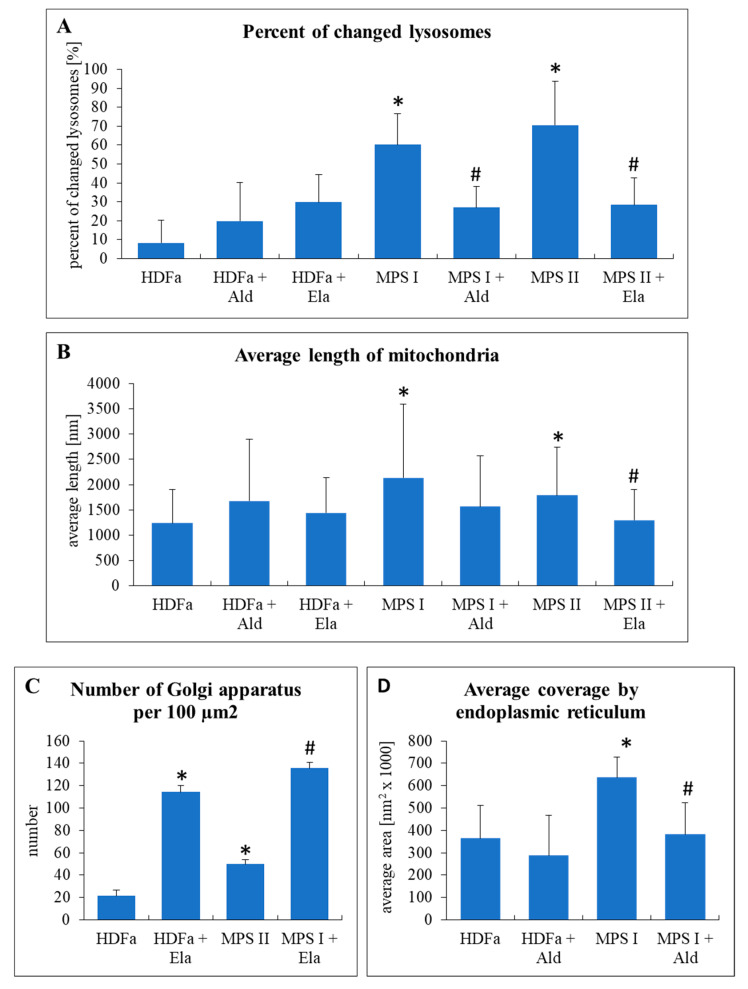
Morphology of lysosomes (**A**), mitochondria (**B**), Golgi apparatus (**C**), and endoplasmic reticulum (**D**) in MPS fibroblasts in comparison to control cells (HDFa cell line) with and without treatment with 0.58 mg/L α-L-iduronidase (Aldurazyme, Ald) or 0.5 mg/L iduronate sulfatase (Elaprase, Ela) for 24 h. Mean values ± SD are presented, with asterisks and hashtags representing statistically significant (*p* < 0.05) differences relative to the HDFa control and untreated MPS, respectively.

Knowing that GAG levels could be corrected by recombinant enzymes, we tested changes in organelles in MPS I and MPS II fibroblasts under these conditions. Electron micrographs were analyzed as in experiments without enzymes. As expected, number of changed lysosomes, which was significantly higher in untreated MPS I and MPS II cells, could be significantly reduced after treatment with α-L-iduronidase and iduronate sulfatase, respectively (Figure 11A). After such a treatment, the differences between MPS and HDFa cells were not statistically significant. Similar phenomenon was observed when considering length of mitochondria (Figure 11B). Interestingly, treatment with iduronate sulfatase caused a significant increase in abundance of Golgi apparatus in both control (HDFa) cells and MPS II fibroblasts (Golgi apparatus was not changed in MPS I, thus, effects of α-L-iduronidase were not tested) (Figure 11C). When testing endoplasmic reticulum, we found that treatment of MPS I fibroblasts with α-L-iduronidase normalized average coverage by this cellular structure, while this enzyme had no effects on HDFa cells (endoplasmic reticulum was not changed in MPS II, thus, effects of iduronate sulfatase were not tested) (Figure 11D).

**Figure 12:**
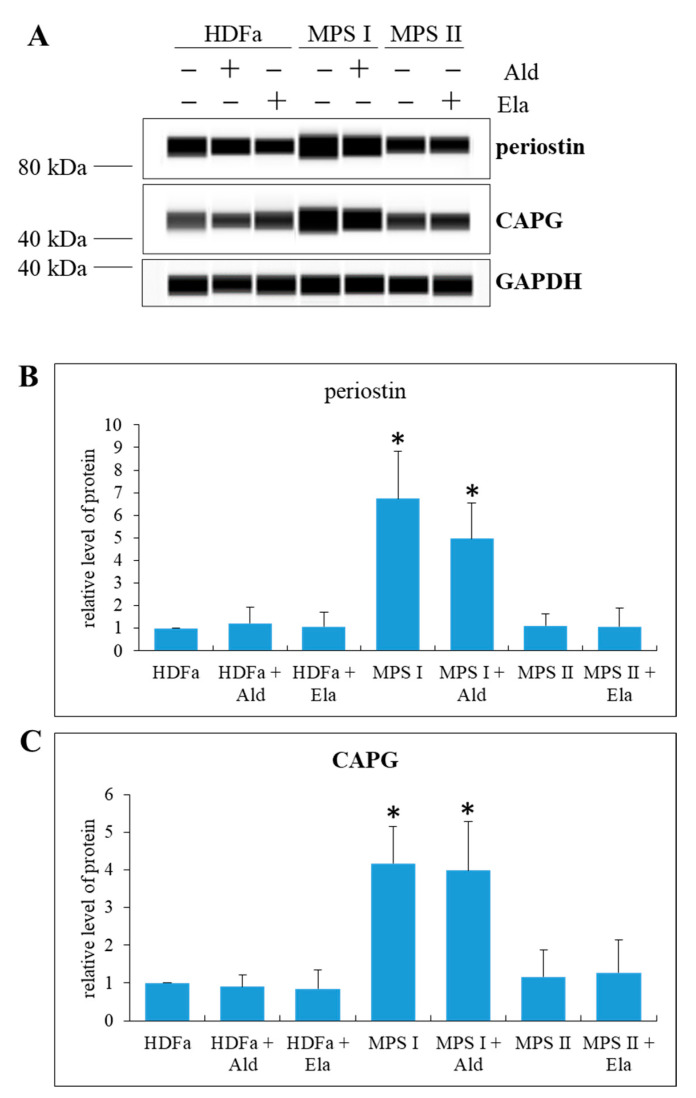
Levels of periostin and CAPG proteins in MPS fibroblasts in comparison to control cells (HDFa cell line) with and without treatment with 0.58 mg/L α-L-iduronidase (Aldurazyme, Ald) or 0.5 mg/L iduronate sulfatase (Elaprase, Ela) for 24 h. Representative Western-blots are demonstrated in (**A**), while quantification of relative levels (assuming 1 as the value in untreated HFDa cells) of periostin and CAPG are shown in (**B**,**C**), respectively. Mean values ± SD are presented, with asterisks and hashtags representing statistically significant (*p* < 0.05) differences relative to the HDFa control and untreated MPS, respectively.

We also asked if enzyme-mediated correction of GAG storage can improve levels of proteins which were found to be significantly changed in MPS fibroblasts. Considering transcriptomic analyses, we chose two proteins, periostin and CAPG, whose genes were significantly upregulated in MPS I but not in MPS II. We confirmed that effects observed at the level of transcripts corresponded to those determined at the level of proteins, namely, periostin and CAPG proteins were significantly more abundant in MPS I, but not in MPS II, relative to HDFa cells (Figure 12). Perhaps surprisingly, we found that treatment of MPS I fibroblasts with α-L-iduronidase, although correcting GAG levels (Figure 10) and morphology of lysosomes, mitochondria, and endoplasmic reticulum (Figure 11), was inefficient in reducing abundance of periostin and CAPG proteins (Figure 12). These results indicate that while eliminating the primary cause of MPS, i.e., elevated GAG levels, may lead to improvement of morphology of at least some cellular organelles, other cellular dysfunctions, like dysregulation of expression of certain genes, might remain unchanged despite the use of recombinant enzyme which is deficient in affected cells.

## 3. Discussion

Although the primary cause of MPS is accumulation of GAGs in lysosomes, it is evident that further changes in cell physiology play crucial roles in appearance of cellular dysfunctions and development of the disease symptoms [4,5,7,8,9,10,11]. Although changes in cellular organelles were reported previously in certain MPS types (for summary, see [4]), no studies were reported to date which would address global changes in these cell components in all MPS types. Therefore, in this study we asked what genes that are important for structures and functions of organelles are expressed differentially in cells derived from patients suffering from all known MPS types and subtypes relative to control cells, and what are qualitative and quantitative changes in organelles in such an experimental system.

When analyzing transcriptomic data, we found that there were significant changes in expression of many genes coding for proteins involved in structures and functions of various organelles. They included between 109 and 322 (depending on MPS type) transcripts covered in the ‘Cellular component organization’ term of the QuickGO database and between 70 and 208 in the ‘Cellular anatomical entity’ term. Importantly, levels of some transcripts were significantly changed in most MPS types, suggesting that there are common mechanisms leading to cellular dysfunctions for the whole group of MPS diseases. This hypothesis may be corroborated by similar changes in organelles, observed under electron microscope, which occurred in all or most MPS types. 

The most pronounced changes in all types of MPS were observed in lysosomes which cannot be a surprise for lysosomal storage diseases. However, we also observed a statistically significant decrease in the size of nucleus in MPS IVA and VI. Such changes might suggest considerable problems with either regulation of gene expression or cell cycle/cell division processes or both, as discussed recently [13]. Moreover, significant changes in both number and size of mitochondria were found in 8 out of 11 types/subtypes of MPS. This may indicate problems with cellular energetic processes, affecting functions of cells and tissues, and leading to various disease symptoms, as summarized by others [14]. In fact, various energy-related problems are common in MPS [4]. Like in mitochondria, changes in abundance and structure or size of Golgi apparatus were common in several MPS types. They include higher or lower abundance (depending on MPS type), as well as changes defined as conversion of Golgi ribbon to Golgi stacks or loss of both Golgi ribbon and integrity of Golgi stacks [15], represented in our analyses by decreased length of ribbons and increased or decreased width of cisterns. Again, such changes may suggest dysfunctions of these organelles. Finally, we observed increased (in MPS I and IIIB) or decreased (in MSP IIID and VII) abundance of endoplasmic reticulum. 

Considering results of both types of analyses presented in this report, transcriptomic studies, and electron microscopic experiments, we propose that one might consider a scenario in which changes is structures (and then, in functions) of organelles (other than lysosomes which are changed due to primary GAG storage and secondary storage of other compounds) in MPS are caused by dysregulation of expression of genes coding for proteins related to them. Such a hypothesis might be corroborated by analyses of genes whose expression is changed in many MPS types and/or is changed particularly strongly, which are indicated in Table 2 and Table 3. For example, the *CAPG* gene product was proposed to be involved in forming nuclear structures [16], and we found that it is overexpressed in most MPS types (Table 2) while changes in cell nuclei were observed in at least two MPS types (Figure 6). The protein encoded by *SAR1A* in involved in the control of transport between endoplasmic reticulum and Golgi apparatus and can influence structures and functions of these organelles [17]. Expression of *SAR1A* was downregulated in the vast majority of MPS cell lines (Table 2), and significant changes in Golgi apparatus and endoplasmic reticulum were evident in several MPS types (Figure 8 and Figure 9). Communication between mitochondria and cytoplasm, which significantly influences mitochondrial structure and functions, is mediated, between others, by phosphor-regulation. One of proteins involved in this regulation is the SH3 binding protein 5 (SH3BP5) [18]. Expression of the gene coding for this protein is upregulated in fibroblasts of all MPS types (Table 2), and significant changes in mitochondrial morphology occurred in most investigated MPS cell lines (Figure 7). These are examples of genes whose dysregulated expression might directly influence changes in structures and functions of various organelles in MPS cells. One should also note that among transcripts listed in Table 2 and Table 3 there are several genes encoding various regulators and other control-related proteins which can indirectly, but significantly, influence different organelles. Finally, Table 2 and Table 3 include only genes whose expression was either significantly changed in all/most MPS types or particularly (over 16-times) changed in some MPS types. Other products of genes included in GO terms ‘Cellular component organization’ and ‘Cellular anatomical entity’, whose expression is affected in different MPS types (Table 1), may also considerably influence various organelles.

Interestingly, we found that although normalization of levels of GAGs in MPS cells, achieved after treatment of MPS I and MPS II cell cultures with recombinant human a-L-iduronidase and iduronate sulfatase, respectively (Figure 10), resulted in improvement in morphology of lysosomes, mitochondria and endoplasmic reticulum, abundance of Golgi apparatus was not corrected (Figure 11). One might hypothesize that this could arise from activation of Golgi apparatus by the presence of the recombinant enzyme itself, rather than from a lack of response to GAG level reduction, as increased number of Golgi apparatus was evident also in control (HDFa) cells treated with iduronate sulfatase (Figure 11C). However, it is difficult to explain another observation, indicating that significant upregulation of expression of genes coding for periostin and CAPG in MPS I could not be reverted by treatment of cells with a-L-iduronidase (Figure 12). Definitely, enzyme-mediated correction of GAG levels can improve some, but not all cellular functions, as dysregulation of important genes may result in various changes in cell physiology even in the absence of GAG storage. One might speculate that such irreversible changes in gene expression regulation can explain a failure of enzyme replacement therapy to correct all MPS symptoms, especially if the treatment is initiated at advanced stages of the disease, the phenomenon that was described previously [5,6].

An obvious limitation of this study is the use of only one cell line per MPS type/subtype. Nevertheless, this was the ‘cost’ of investigating all known MPS types simultaneously. Moreover, since some common patterns in gene expression (Table 2 and Table 3) and organelles’ dysmorphology (Figure 5, Figure 6, Figure 7, Figure 8 and Figure 9) were observed, the detected changes should be considered reliable rather than random. In fact, reliability of studies based on the use of the same set of cell cultures has been indicated previously [8,9,10,11,19]. Another question can be asked whether fibroblasts are a good model, reflecting changes in MPS. Although changes in skin structure and function are evident in all MPS types [4], they do not cause the most severe symptoms of the disease. Obviously, testing other types of cells would be profitable, however, one should note that the biological material comes from patients which are severely affected children, thus, obtaining other types of cells is extremely difficult due to both technical and ethical reasons. In fact, fibroblasts have been considered standard cellular models in studies on MPS for many years, irrespective of their limitations in interpretation of experimental data [4]. 

## 4. Materials and Methods

### 4.1. Fibroblast Lines

Lines of fibroblasts derived from patients suffering from all known MPS types (I, II, IIIA, IIIB, IIIC, IIID, IVA, IVB, VI, VII, and IX), described previously [8,9] and purchased from the NIGMS Human Genetic Cell Repository at the Coriell Institute for Medical Research (this institute possess all documents concerning bio-ethical issues) were employed. In control experiments, HDFa (Human Dermal Fibroblast, adult) cell line was used. Characteristics of all cell lines used in this work, with indicated mutations, is provided in Supplementary Table S2.

### 4.2. Cell Cultures

Fibroblasts were cultured using DMEM medium, supplemented with antibiotics and 10% fetal bovine serum (FBS). All cultures used in experiments were between 4th and 15th passage. The cultures were incubated at 37 °C, 95% humidity, and atmosphere saturated with 5% CO_2_. When indicated, the medium was supplemented with human recombinant enzyme, either 0.58 mg/L of a-L-iduronidase (Aldurazyme (laronidase), Genzyme, Cambride, MA, USA) or 0.5 mg/L of iduronate sulfatase (Elaprase (idursulfase), Shire Human Genetic Therapies Inc., Lexington, MA, USA), for 24 h.

### 4.3. RNA-Seq Analysis

The raw data of RNA-seq analysis have been obtained previously [8,9], and deposited in the NCBI Sequence Read Archive (SRA), under accession no. PRJNA562649mRNA. Reliability of the transcriptomic data was confirmed by measurement of mRNA levels using RT-qPCR [8,9], and determination of either activities [10] or levels [11] of selected gene products. The GTF Homo_sapiens.GRCh38.94.gtf file from the Ensembl database and the Cuffquant and Cuffmerge programs (version 2.2.1) were employed to calculate the levels of transcripts. The Cuffmerge program was started with the library-norm-method classic-fpkm parameter normalizing the expression values by means of the FPKM algorithm. Transcripts of genes included into terms ‘Cellular component organization’ (GO:0016043) and ‘Cellular anatomical entity’ (GO:0110165) of the QuickGO database were analyzed at false discovery rate (FDR) < 0.1 and *p* < 0.1, according to previous publications [8,9,10,11].

### 4.4. Electron Microscopic Analyses

Samples for electron microscopic analyses were prepared as described previously [12]. Microscopic analyses were performed using the Philips CM100 microscope. Electron micrographs were used to assess morphologies of different cellular organelles. At least 100 cells were considered in each case. Depending on assessed cellular structures, their dimensions and/or abundance in cells were measured, calculated, and analyzed, considering differences between control cells (HDFa line) and fibroblasts derived from patients suffering from all known types and subtypes. 

### 4.5. Determination of GAG Levels in Cells

Levels of GAGs in cultured fibroblasts were determined using the Glycosaminoglycan Assay Blyscan^TM^ (Biocolor Ltd., Carrickfergus, UK), according to manufacturer’s instruction. 

### 4.6. Western-Blotting Analyses

Cells (6 × 10^5^) were seeded on 10 cm-diameter plates and allowed to attach overnight under conditions described in Section 4.2. To lyse the cells, the solution composed of 1% Triton X-100, 0.5 mM EDTA, 150 mM NaCl, 50 mM Tris, 274 pH 7.5, and a mixture of protease and phosphatase inhibitors (Roche Applied Science, Penzberg, Germany) were used. Following clarification by centrifugation in a microcentrifuge, proteins were separated and detected using the WES system (WES—Automated Western 277 Blots with Simple Western; ProteinSimple, San Jose, CA, USA), with 12–230 kDa Separation Module and Anti-Mouse Detection Module, as described by the manufacturer. Anti-periostin antibodies (Abcam, Cambridge, UK), anti-CAPG antibodies (Abcam, Cambridge, UK), and monoclonal anti-GAPDH-peroxidase antibody (Merck, Darmstadt, Germany) were used for detection of specific proteins.

### 4.7. Statistical Analyses

One-way ANOVA on log_2_(1 + x) values which have normal continuous distribution was used for statistical significance assessment. The Benjamini–Hochberg method was used to calculate the false discovery rate (FDR). Post hoc Student’s *t*-test with Bonferroni correction was used for comparisons between two groups. 

## 5. Conclusions

Significant changes in expression of hundreds of genes related to structures and functions of cellular organelles suggests that dysregulation of these genes significantly contributes to dysmorphology of lysosomes, nuclei, mitochondria, Golgi apparatus, and endoplasmic reticulum in MPS cells. Some, but not all, changes can be corrected by enzyme-mediated normalization of GAG levels. This might underline significance of disturbed control of gene expression, resulting in accumulation of defects in organelles, in pathomechanisms of all types of MPS. 

## Figures and Tables

**Table 1:** Number of up- and downregulated transcripts (at FDR < 0.1; *p* < 0.1) of genes included into GO terms ‘Cellular component organization’ (GO:0016043) and ‘Cellular anatomical entity’ (GO:0110165) in different types of MPS relative to control cells (HDFa).

Transcripts	No. of Transcripts with Changed Expression Levels in Each MPS Type vs. HDFa Line										
	I	II	IIIA	IIIB	IIIC	IIID	IVA	IVB	VI	VII	IX
	Cellular component organization (GO:0016043)										
Upregulated	123	79	139	145	124	114	49	162	68	131	129
Downregulated	139	54	183	159	126	147	73	151	41	156	183
Total	262	133	322	304	250	261	122	313	109	287	312
	Cellular anatomical entity (GO:0110165)										
Upregulated	75	54	75	90	86	57	38	93	37	60	83
Downregulated	96	41	125	104	99	85	45	115	33	98	109
Total	171	95	200	194	185	142	83	208	70	158	192

**Table 2:** Genes included into GO terms ‘Cellular component organization’ (GO:0016043) and ‘Cellular anatomical entity’ (GO:0110165) whose transcript levels were of up- (red) and down- (blue) regulated (at FDR < 0.1; *p* < 0.1) in at least nine (out of 11) types/subtypes of MPS relative to control cells (HDFa). Fold change (FC) levels are presented for individual transcripts. Noncolored boxes in the table indicate a lack of significance in the statistical analyses of differences between MPS and control cell lines.

GO Term/Gene	log_2_FC of Transcripts whose Levels Were Changed in Most (at Least Nine) MPS Types vs. HDFa Line										
Cellular Component organization	I	II	IIIA	IIIB	IIIC	IIID	IVA	IVB	VI	VII	IX
*MFAP5*	3.50	5.49	6.14	5.00	4.51	3.75	3.93	4.11	4.61	4.20	4.40
*PFN1*	3.45	3.71	3.51	3.64	3.73	3.53	3.62	3.67	3.50	3.67	2.94
*CAPG*	2.73	2.98	4.71	1.98	4.27	2.75	4.17	4.14	3.82	4.28	4.55
*POSTN*	4.38	4.27	5.27	7.26	5.42	5.01	3.70	5.69	5.47	5.20	5.83
*SIN3B*	−1.18	−1.55	−1.24	−1.47	−1.61	−1.92	−1.52	−1.69	−1.57	−1.67	−1.67
*SAR1A*	−0.61	−0.55	−0.68	−0.61	−0.69	−0.31	−0.74	−0.64	−0.46	−0.46	−0.48
Cellular anatomical entity	I	II	IIIA	IIIB	IIIC	IIID	IVA	IVB	VI	VII	IX
*SH3BP5*	1.37	1.34	1.76	1.38	1.08	0.63	0.86	1.02	1.29	1.24	0.96
*POSTN*	4.38	4.27	5.27	7.26	5.42	5.01	3.70	5.69	5.47	5.20	5.83
*ARSA*	1.06	1.13	1.13	1.09	0.14	1.48	1.00	0.80	0.92	1.36	1.11
*TMEM38B*	−0.90	−0.87	−1.34	−1.06	−1.12	−1.44	−1.09	−0.86	−0.47	−1.18	−0.99
*EMP1*	−3.38	−3.43	−3.42	−3.67	−4.71	−3.11	−4.09	−3.74	−2.45	−1.79	−3.09
*ABHD5*	−1.69	−1.23	−1.74	−1.04	−1.08	−0.66	−1.22	−0.77	−0.99	−1.53	−1.06

**Table 3:** Genes with log_2_FC > 4.0 in specific MPS type relative to control cells (HDFa). Up-arrowed and down-arrowed symbols indicate up- and downregulation, respectively, while minus symbols indicate no significant changes.

GO Term/Gene	Extremely Changed Transcripts (log_2_FC > 4) in Particular MPS Type vs. HDFa Line										
Cellular Component Organization	I	II	IIIA	IIIB	IIIC	IIID	IVA	IVB	VI	VII	IX
*TNFRSF11B*	-	-	-	-	-	-	-	-	-	↓	-
*CAV1*	↓	-	-	-	-	-	-	↓	-	-	-
*WISP2*	-	-	↓	↓	-	-	-	-	-	-	-
*APOE*	-	-	↓	-	-	-	-	-	-	-	-
*SNX3*	-	-	-	-	↓	-	-	-	-	-	-
*SPON2*	-	-	↓	-	-	-	-	-	-	-	-
*WISP2*	-	-	-	↓	-	-	-	-	-	-	-
*MMP12*	-	-	↓	↓	↓	-	-	-	-	-	↓
*MMP3*	-	-	-	-	-	-	-	-	-	-	↓
*IGFBP5*	-	-	-	-	-	↑	-	-	-	-	↑
*COMP*	-	-	-	-	-	↑	-	-	-	-	-
*COL8A2*	-	-	-	-	↑	↑	-	-	-	-	-
*CD9*	-	-	-	↑	-	-	-	↑	-	-	-
*CAPG*	-	-	↑	-	↑	-	↑	↑	-	↑	↑
*POSTN*	↑	-	↑	↑	↑	↑	-	↑	↑	↑	↑
*POSTN*	↑	-	↑	↑	↑	↑	-	↑	↑	-	↑
*POSTN*	↑	-	↑	↑	-	↑	-	↑	↑	↑	↑
*KRT19*	-	-	-	-	-	-	-	↑	-	-	-
*OXTR*	-	-	-	↑	↑	↑	-	↑	-	-	↑
*MFAP5*	-	↑	↑	↑	↑	-	-	↑	↑	↑	↑
Cellular anatomical entity	I	II	IIIA	IIIB	IIIC	IIID	IVA	IVB	VI	VII	IX
*EMP1*	-	-	-	-	↓	-	↓	-	-	-	-
*RARRES2*	-	-	↓	↓	-	-	-	-	-	-	-
*PTGS1*	-	-	-	-	-	-	-	-	-	↓	-
*APOE*	-	-	↓	-	-	-	-	-	-	-	-
*THBS1*	-	↓	-	-	-	-	-	↓	-	-	-
*GNS*	-	-	-	-	-	↓	-	-	-	-	-
*RPL23*	-	-	-	-	↓	↓	↓	-	-	-	-
*PTGS1*	↓	-	-	-	-	-	-	-	-	-	-
*PTGS1*	-	-	-	-	-	-	-	-	-	↓	-
*PTGDS*	-	-	↓	↓	↓	-	-	↓	-	↓	↓
*EMP1*	-	-	-	-	↓	-	↓	-	-	-	-
*CAV1*	↓	-	-	-	-	-	-	↓	-	-	-
*RPL10*	-	-	-	-	-	↓	-	-	-	-	-
*POSTN*	↑	-	↑	↑	↑	↑	-	↑	↑	↑	↑
*POSTN*	↑	-	↑	↑	↑	↑	-	↑	↑	-	↑
*POSTN*	↑	-	↑	↑	-	↑	-	↑	↑	-	-
*CD9*	-	-	-	↑	-	-	-	↑	-	-	-
*MFGE8*	-	-	↑	-	-	-	-	-	-	-	-

## Data Availability

RNA-seq raw results are deposited in the NCBI Sequence Read Archive (SRA), under accession no. PRJNA562649mRNA. Other raw results are available from the authors upon request.

## Supplementary Materials

The following are available online at https://www.mdpi.com/1422-0067/22/5/2766/s1.
