# Supplementary material for: Gene Expression-Related Changes in Morphologies of Organelles and Cellular Component Organization in Mucopolysaccharidoses
Source: Int J Mol Sci. 2021 Mar 9;22(5):2766. doi: 10.3390/ijms22052766 (PMC7967209; doi:10.3390/ijms22052766)
Supplement: Supplementary file 1 [file ijms-22-02766-s001.zip › ijms-1075821-REVISED-SUPPLEMENTARY-MATERIAL-FINAL.pdf]

SUPPLEMENTARY MATERIAL

Table S1. Classification of MPS types.

MPS Type	OMIM no.		Deficient enzyme		GAG(s) stored in cells[a]
	PMIM	Gene-locus MIM	Name	EC no.	
**I**	607014, 607015, 607016	252800	α-L-iduronidase	3.2.1.76	HS, DS
II	309900	300823	2-iduronate sulfatase	3.1.6.13	HS, DS
IIIA	252900	605270	N-sulfoglucosamine sulfhydrolase	3.10.1.1	HS (secondary storage of DS)
IIIB	252920	609701	α-N-acetylglucosaminidase	3.2.1.50	HS (secondary storage of DS)
IIIC	252930	610453	Acetyl-CoA:α-glucosaminide acetyltransferase	2.3.1.78	HS (secondary storage of DS)
IIID	252940	607664	N-acetylglucosamine 6-sulfatase	3.1.6.14	HS (secondary storage of DS)
IVA	253000	612222	N-acetylgalactosamine 6-sulfatase	3.1.6.4	KS, CS
IVB	253010	611458	β-galactosidase-1	3.2.1.23	KS
VI	253200	611542	N-acetylgalactosamine 4-sulfatase	3.1.6.12	DS
VII	253220	611499	β-glucuronidase	3.2.1.31	HS, DS, CS
IX	601492	607071	Hyaluronidase-1	3.2.1.35	HA

[a]Abbreviations: CS, chondroitin sulfate; DS, dermatan sulfate; HA, hyaluronic acid; HS, heparan sulfate; KS, keratan sulfate.

Table S2. Characteristics of fibroblast lines used in this work.

MPS type	Mutated gene and its locus	Mutation(s) in the used fibroblast line[a]	Catalog number of the cell line[b]
MPS I	*IDUA*, 4p16.3	p.Trp402Ter/p.Trp402Ter	GM00798
MPS II	*IDS*, Xp28	p.His70ProfsTer29	GM13203
MPS IIIA	*SGSH*, 17q25.3	p.Glu447Lys/p.Arg245His	GM00879
MPS IIIB	*NAGLU*, 17q21	p.Arg626Ter/p.Arg626Ter	GM00156
MPS IIIC	*HGSNAT*, 8p11.1	Not determined	GM05157
MPS IIID	*GNS*, 12q14	p.Arg355Ter/p.Arg355Ter	GM05093
MPS IVA	*GALNS*, 16q24.3	Not determined	GM00593
MPS IVB	*GLB1*, 3p22.3	p.Trp273Leu/p.Trp509Cys	GM03251
MPS VI	*ARSB*, 4q14.1	Not determined	GM03722
MPS VII	*GUSB*, 7q21.11	p.Trp627Cys/p.Arg356Ter	GM00121
MPS IX	*HYAL1*, 3p.21.3	Not determined	GM17494
Control line (HDFa)	N/A	N/A	N/A

(a) When mutations were not determined, the diagnosis of specific MPS type was based on analysis of urinary GAG levels, with indication of kind(s) of GAG(s) with elevated amounts in tested samples, and biochemical determination of deficiency of particular lysosomal enzyme in leukocytes; phenotypes of all patients were severe, according to available data. (b) Catalog numbers are according to cell line description in NIGMS Human Genetic Cell Repository at the Coriell Institute for Medical Research.

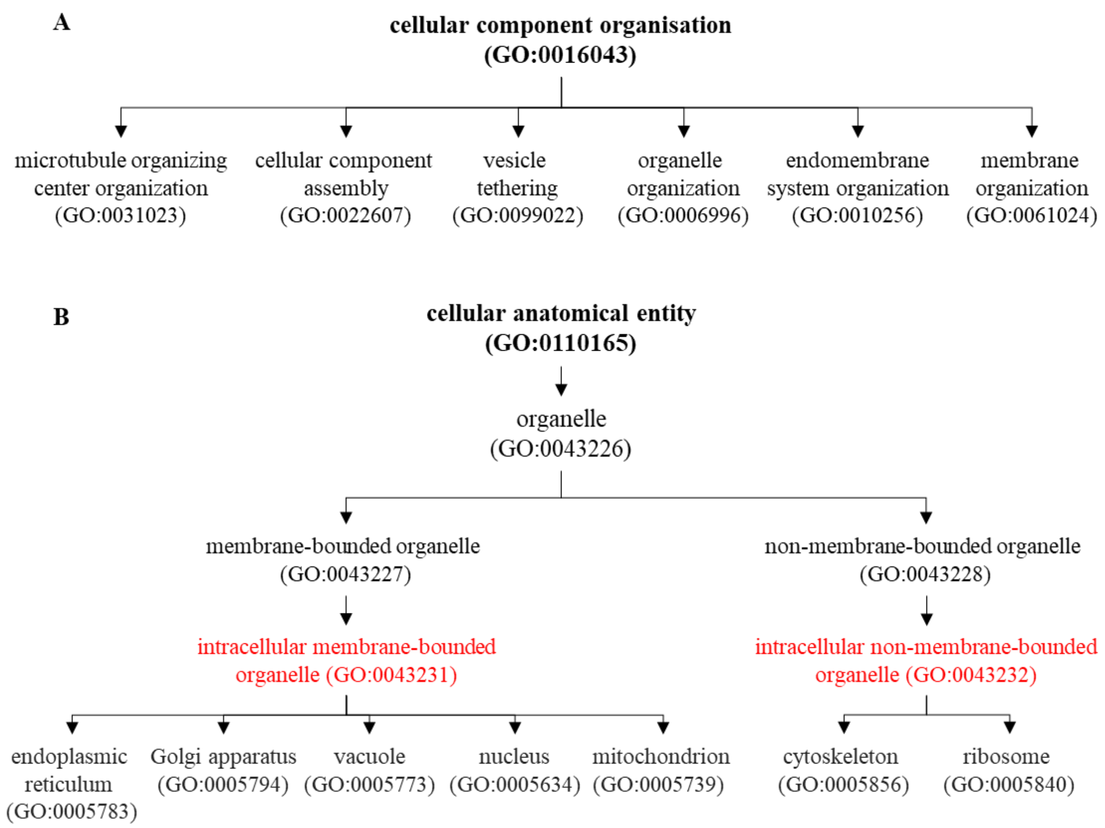

Figure S1. Child terms (with GO numbers) of 'cellular component organization' (GO:0016043) (A) and 'cellular anatomical entity' (GO:0110165) (B) terms, considered in detailed transcriptomic analyzes performed in this study.
